# *PEPC* of sugarcane regulated glutathione S-transferase and altered carbon–nitrogen metabolism under different N source concentrations in *Oryza sativa*

**DOI:** 10.1186/s12870-021-03071-w

**Published:** 2021-06-24

**Authors:** Ling Lian, Yuelong Lin, Yidong Wei, Wei He, Qiuhua Cai, Wei Huang, Yanmei Zheng, Huibin Xu, Fuxiang Wang, Yongsheng Zhu, Xi Luo, Huaan Xie, Jianfu Zhang

**Affiliations:** 1 Rice Research Institute, Fujian Academy of Agricultural Sciences, 350019 Fuzhou, Fujian China; 2 State Key Laboratory of Ecological Pest Control for Fujian and Taiwan Crops, Key Laboratory of Germplasm Innovation and Molecular Breeding of Hybrid Rice for South China, Ministry of Agriculture/South-China Base of National Key Laboratory of Hybrid Rice of China/National Engineering Laboratory of Rice, Fujian Academy of Agricultural Sciences, 350003 Fuzhou, Fujian China; 3 Institute of Quality Standards & Testing Technology for Agro-Products, Fujian Academy of Agricultural Sciences, 350003 Fuzhou, Fujian China

**Keywords:** PEPC, Nitrogen-carbohydrate metabolism, Proteomic analysis, Gene expression, Enzyme activity, Phytohormone content

## Abstract

**Background:**

Phosphoenolpyruvate carboxylase (PEPC) plays an important role in the primary metabolism of higher plants. Several studies have revealed the critical importance of PEPC in the interaction of carbon and nitrogen metabolism. However, the function mechanism of PEPC in nitrogen metabolism is unclear and needs further investigation.

**Results:**

This study indicates that transgenic rice expressing the sugarcane *C4-PEPC* gene displayed shorter primary roots and fewer crown roots at the seedling stage. However, total nitrogen content was significantly higher in transgenic rice than in wild type (WT) plants. Proteomic analysis revealed that there were more differentially expressed proteins (DEPs) responding to nitrogen changes in transgenic rice. In particular, the most enriched pathway “glutathione (GSH) metabolism”, which mainly contains GSH S-transferase (GST), was identified in transgenic rice. The expression of endogenous *PEPC*, *GST* and several genes involved in the TCA cycle, glycolysis and nitrogen assimilation changed in transgenic rice. Correspondingly, the activity of enzymes including GST, citrate synthase, 6-phosphofructokinase, pyruvate kinase and ferredoxin-dependent glutamate synthase significantly changed. In addition, the levels of organic acids in the TCA cycle and carbohydrates including sucrose, starch and soluble sugar altered in transgenic rice under different nitrogen source concentrations. GSH that the substrate of GST and its components including glutamic acid, cysteine and glycine accumulated in transgenic rice. Moreover, the levels of phytohormones including indoleacetic acid (IAA), zeatin (ZT) and isopentenyladenosine (2ip) were lower in the roots of transgenic rice under total nutrients. Taken together, the phenotype, physiological and biochemical characteristics of transgenic rice expressing *C*_*4*_*-PEPC* were different from WT under different nitrogen levels.

**Conclusions:**

Our results revealed the possibility that PEPC affects nitrogen metabolism through regulating GST, which provide a new direction and concepts for the further study of the PEPC functional mechanism in nitrogen metabolism.

**Supplementary Information:**

The online version contains supplementary material available at 10.1186/s12870-021-03071-w.

## Background

PEPC widely exists in vascular plants, cyanobacteria, green algae, non-photosynthetic bacteria and archaea, but not in fungi and animals [1, 2]. PEPC is recognized for its special role in the carbon metabolism of plants. In C_4_ and crassulaceae plants, PEPC plays a critical role in the initial fixation of atmospheric CO_2_ in C_4_ photosynthesis and crassulacean acid metabolism photosynthesis. In C_3_ plants and most non-photosynthetic tissues, PEPC primarily catalyzes irreversible β-carboxylation of phosphoenolpyruvate (PEP) in the presence of HCO_3_^−^ and Me^2+^ to form oxaloacetate (OAA) and inorganic phosphate (Pi) [1]. OAA is the carbon skeleton for the synthesis of aspartic acid (Asp) and asparagine (Asn), and an important material in the tricarboxylic acid (TCA) cycle, which generates intermediates involved in other physiological functions and provides carbon skeletons for biosynthesis and nitrogen assimilation [2].

PEPC is an abundant plant protein that is used for nitrogen storage [3]. Moreover, PEPC has an important role in carbon and nitrogen interactions [4]. Knockdown of *Osppc4* encoding a plant-type PEPC targeted to chloroplasts in rice resulted in a decrease of OAA, citric acid and isocitric acid. Meanwhile, glutamate and aspartate contents decreased, and the nitrogen content of the whole plant is reduced in *Osppc4* knockdown lines, which indicates that the down-regulation of *Osppc4* suppresses NH_4_^+^ assimilation and amino acid synthesis [5]. A double mutant of *PPC1* and *PPC2* encoding PEPC in Arabidopsis showed a growth-arrest phenotype and decreased PEPC activity, and further reduced the synthesis of malate and citrate and severely suppressed ammonium assimilation [6]. Transgenic rice overexpressing maize *PEPC* had increased carbon levels, higher grain yield per plant and were more tolerant to low nitrogen stress with up-regulation of photorespiratory genes [7]. Overexpression of *Zea mays PEPC* in *Arabidopsis thaliana* resulted in increasing protein and free amino acid content in transgenic plants [8]. Seed-specific expression of a bacterial *PEPC* in *Vicia narbonensis* resulted in higher seed-dry weight, elevating crude protein and free amino acids content [9]. These studies suggested that a change in *PEPC* expression in plants altered physiology and biochemistry with changes of biomass, organic acid content, protein and amino acid levels. In addition, Dof1 transcription factor regulated the expression of genes, such as *PEPC*, to modulate carbon and nitrogen metabolites. The expression of maize *Dof1* in *Arabidopsis thaliana* and rice significantly enhanced *PEPC* gene expression, increased PEPC activity and amino acid content and improved growth under low nitrogen conditions [10, 11]. The research indirectly proves the function of PEPC in carbon and nitrogen metabolism. Moreover, overexpression of *GS1;1* and *GS1;2* encoding glutamine synthetase, a key enzyme involved in ammonium assimilation, induced a decrease of *PEPC* genes expression [12], and glutamine synthetase 2 co-suppressed plants showed a significant decrease of *PEPC1* and *PEPC2* gene expression [13], which suggested that *PEPC* was associated with nitrogen metabolism. PEPC is involved in nitrogen metabolism, but it may not directly participate in nitrogen assimilation. The effects of PEPC in the modulation of carbon and nitrogen interactions need to be further clarified.

In this study, the sugarcane *C*_*4*_*-PEPC* gene was introduced into the indica cultivar Hang2. To further investigate phenotype and physiological-biochemical changes, transgenic plants and WT (indica rice cultivar, Hang2) plants were cultured in nutrient solution with different concentrations of nitrogen source. Then, proteomic analysis was performed to identify DEPs and the relevant metabolic pathways. Additionally, gene expression, enzyme activities, metabolites and hormones were measured and comparatively analyzed. Finally, we identified DEPs with significant changes in transgenic rice expressing *C*_*4*_*-PEPC*. We also summarized and obtained the differences in the physiology and biochemistry between transgenic rice expressing *C*_*4*_*-PEPC* and WT plants.

## Results

### Plant phenotype under different nitrogen source concentrations

**Fig. 1:**
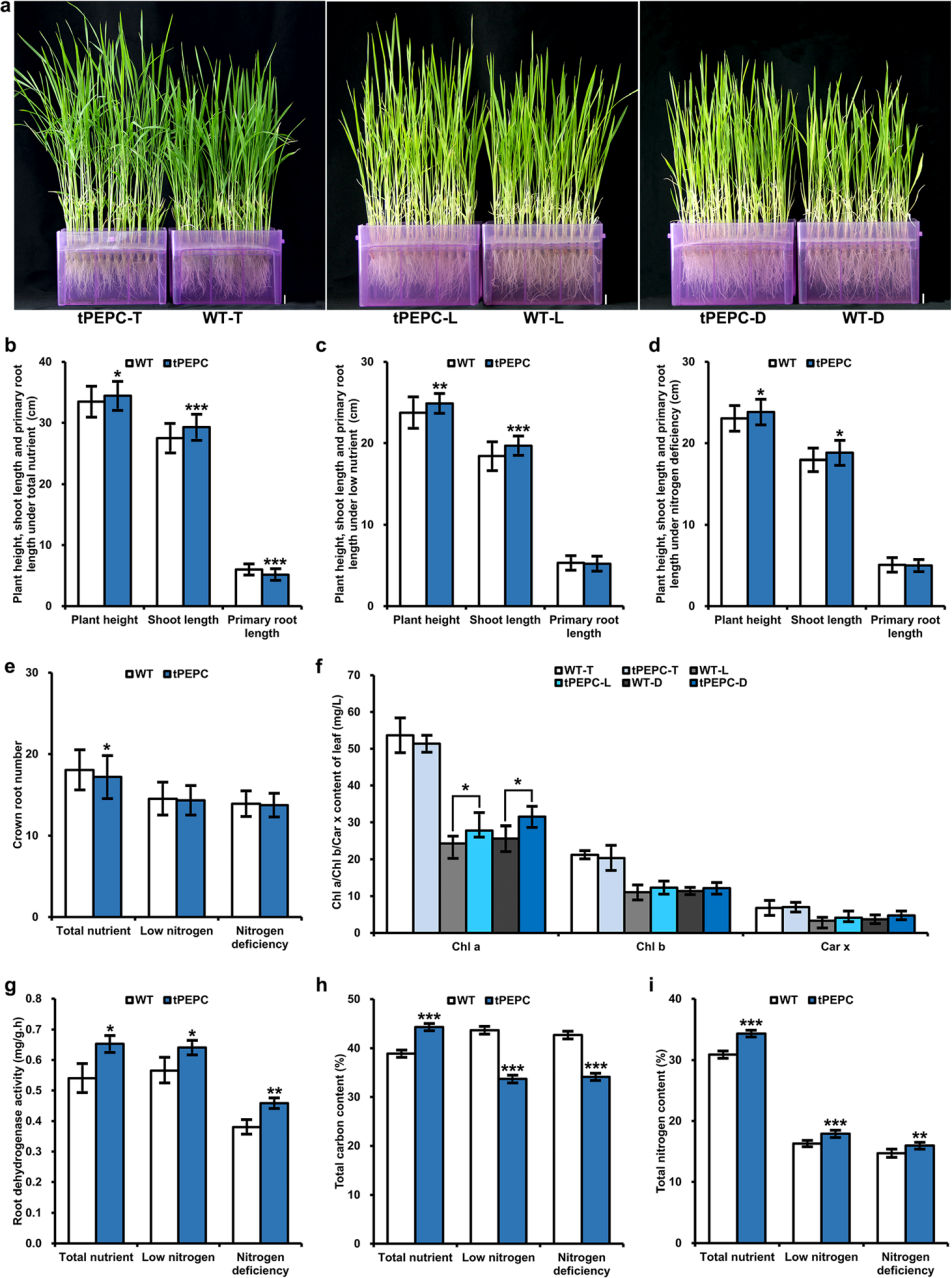
Phenotype of transgenic rice expressing *C4-PEPC* (tPEPC). **a-d** Plants of tPEPC and WT under total nutrients, low nitrogen and nitrogen deficiency. **e** Crown root number. **f** Chlorophyll (Chl) and carotenoid (Car x) content. **g** Root dehydrogenase activity. **h** Total carbon content. **i** Total nitrogen content. Bar = 1 cm. (*P ≤ 0.05, **P ≤ 0.01, ***P ≤ 0.001)

Previously, we introduced the sugarcane *PEPC* gene containing the promoter and coding region into indica cultivar Hang2 using Agrobacterium-mediated transformation and generated tPEPC transgenic lines. The *C*_*4*_*-PEPC* was successfully expressed at the transcriptional and protein level in tPEPC lines. Correspondingly, all tPEPC lines showed higher PEPC enzyme activities compared with WT [14]. Moreover, the total nitrogen content of tPEPC lines increased at both the seedling and tillering stages (Supplementary Figure S1). This suggested that the expression of *C*_*4*_*-PEPC* influenced both carbon metabolism and nitrogen metabolism in transgenic rice. Then we investigated the phenotype and biochemical characteristics of tPEPC and WT plants under different nitrogen source concentrations. The tPEPC and WT plants were cultured in total nutrient solution containing normal levels of nitrogen and labeled as tPEPC-T and WT-T. To investigate the difference of sensitivity to nitrogen, tPEPC and WT plants were cultured in low nitrogen solution containing trace nitrogen and labeled as tPEPC-L and WT-L. To better investigate the growth difference between tPEPC and WT plants, they were also cultured in nitrogen deficiency solution and labeled as tPEPC-D and WT-D. The plant height and shoot length measurements of tPEPC plants were significantly higher than that of WT plants in different conditions. The primary root length of tPEPC plants was shorter than that of WT under total nutrients, while there were no differences between tPEPC and WT plants under low nitrogen and nitrogen deficiency (Fig. 1a-d). Notably, the crown root number of tPEPC plants was lower than that of WT plants under total nutrients (Fig. 1e). The chlorophyll a (Chl a) content of tPEPC-L and tPEPC-D was greater than that of WT-L and WT-D, respectively (Fig. 1f). In addition, tPEPC plants showed higher root dehydrogenase activity (Fig. 1 g). Correspondingly, the Chl a content and root dehydrogenase activity increased in 6-day-old seedlings of tPEPC lines (Supplementary Figure S2). Compared with that of WT, the total carbon content of tPEPC plants increased by about 14 % under total nutrients. By contrast, they decreased by approximately 22 and 20 % under low nitrogen and nitrogen deficiency, respectively (Fig. 1 h). The total nitrogen content of tPEPC plants increased significantly under different nitrogen source concentrations (Fig. 1i). These results suggested that the expression of *C*_*4*_*-PEPC* influenced growth and physiology in *Oryza sativa*.

### Proteomic analysis

**Fig. 2:**
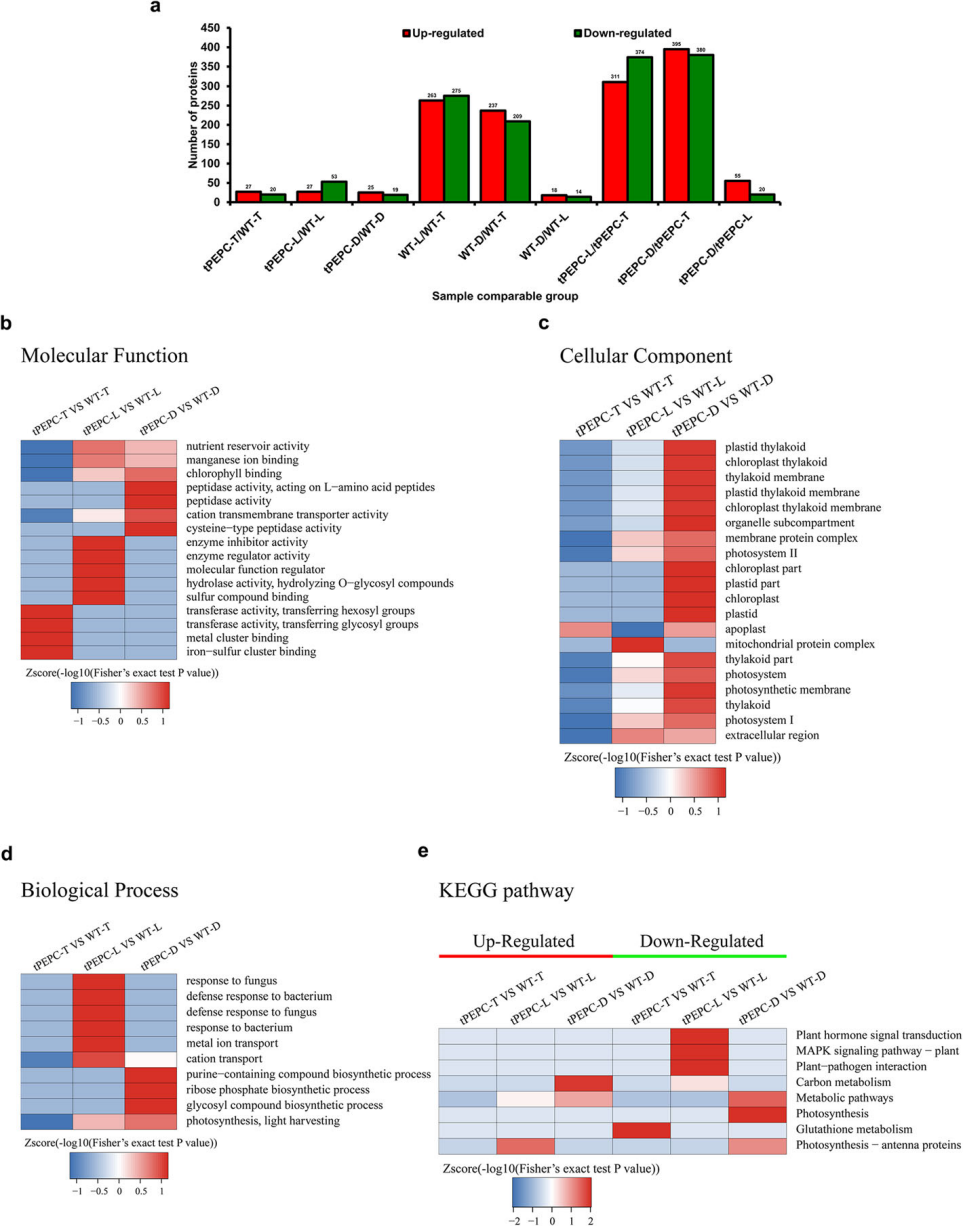
Proteomic analysis. **a** Number of differentially expressed proteins. **b-d** Comparative cluster analysis of GO enriched analysis results. Comparative cluster of **b** molecular function, **c** cellular component, **d** biological process and **e** KEGG pathway

To clarify the molecular mechanisms of C_4_-PEPC regulating plant growth in *Oryza sativa*, we conducted protein profile analysis by the tandem mass tag (TMT) method. Proteins with fold changes (FC) greater than 1.3 and less than 1/1.3 were identified as DEPs. Overall, 685, 775 and 75 DEPs were identified in tPEPC-L/tPEPC-T, tPEPC-D/tPEPC-T, and tPEPC-D/tPEPC-L, respectively, which were more than the 538, 446 and 32 DEPs identified in WT-L/WT-T, WT-D/WT-T, and WT-D/WT-L. This implied that more genes were involved in the response to nitrogen changes in tPEPC plants. Overall, 47 DEPs (27 up-regulated and 20 down-regulated), 80 DEPs (27 up-regulated and 53 down-regulated) and 44 DEPs (25 up-regulated and 19 down-regulated) were identified in tPEPC-T/WT-T, tPEPC-L/WT-L and tPEPC-D/WT-D, respectively (Fig. 2a). Then, we focused on the DEPs identified in tPEPC-T/WT-T, tPEPC-L/WT-L and tPEPC-D/WT-D. DEPs were annotated and classified into the three major Gene Ontology (GO) categories: biological process, cellular component and molecular function. The primary functional GO terms were cellular process and metabolic process in the biological process category; cell, membrane and organelle in the cellular component category; and binding and catalytic activity in the molecular function category (Supplementary Figure S3). The DEPs were localized predominantly in chloroplasts and cytoplasm (Supplementary Figure S4), which implied a change of metabolic activity in tPEPC plants. GO enrichment analysis was performed to identify significant functional classifications. Then, comparative cluster analysis was carried out to indicate a comparison between the groups (Fig. 2b-d, Supplementary Figure S5). In molecular function ontology, the prominent classifications were transferase activity and metal/iron-sulfur cluster in tPEPC-T/WT-T, including enzyme inhibitor/regulator activity, molecular function regulator, hydrolase activity, sulfur compound binding, nutrient reservoir activity and manganese ion binding in tPEPC-L/WT-L and peptidase activity, cation transmembrane transporter activity, cysteine-type peptidase activity and chlorophyll-binding in tPEPC-D/WT-D. In cellular component ontology, the prominent classifications were apoplast in tPEPC-T/WT-T, mitochondrial protein complex and extracellular region in tPEPC-L/WT-L and classed of thylakoid and photosystem in tPEPC-D/WT-D. In biological process ontology, the prominent classifications were cellular response to chemicals/stress in tPEPC-T/WT-T, defense response and metal ion transport in tPEPC-L/WT-L and purine-containing compound/ribose phosphate/glycosyl compound biosynthetic process and photosynthesis in tPEPC-D/WT-D. The results suggested that the nitrogen responsiveness of tPEPC plants significantly differed from WT plants and there was a significant difference between the tPEPC-T/WT-T, tPEPC-L/WT-L and tPEPC-D/WT-D groups.

Subsequently, the enriched pathways were identified in the Kyoto Encyclopedia of Genes and Genomes (KEGG) database and assessed by cluster analysis (Fig. 2e; Table 1, Supplementary Figure S6). In tPEPC-T/WT-T, an up-regulated and a down-regulated pathway “GSH metabolism” was identified and included two GSTs. In this study, tPEPC plants with down-regulated GST had shorter primary roots and fewer crown roots, which was consistent with a loss-of-function rice mutant with a T-DNA insertion in GST showed reduced primary root elongation and lateral root formation [15]. In tPEPC-L/WT-L, the up-regulated pathway category “photosynthesis - antenna proteins” included “light-harvesting II chlorophyll a/b binding protein 1 (Lhcb1)” and “light-harvesting complex I chlorophyll a/b binding protein 2 (Lhca2)”. The down-regulated pathway “plant hormone signal transduction/MAPK signaling pathway–plant/plant-pathogen interaction” included “pathogenesis-related proteins (PRs)”. In tPEPC-D/WT-D, the enriched pathway “carbon metabolism” included up-regulated proteins “isocitrate lyase” and “malic enzyme” (ME), and down-regulated protein “pyruvate phosphate dikinase” (PPDK). The down-regulated pathway “photosynthesis” included “photosystem I reaction center subunit XI, chloroplastic”. The enriched pathway “metabolic pathways/photosynthesis-antenna proteins” included up-regulated “alpha-amylase”, and down-regulated “chlorophyll a-b binding protein, chloroplastic” (Lhcb1) and “lipoxygenase” (LOX). These results indicated that the DEPs in tPEPC-L/WT-L and tPEPC-D/WT-D were mainly related to photosynthesis and carbon metabolism.

**Table 1:** Annotation of KEGG-enrichment pathways

KEGG pathway	Protein coding (Transcript)	Gene ID^*1^	Gene ID in RAP^*2^	Regulation	***P*** value	Annotation
**tPEPC-T VS WT-T**						
osa00480 Glutathione metabolism	Os10t0528300	BGIOSGA033328	Os10g0528300	Down	0.0001848	Glutathione S-transferase, GST4
	Os03t0283200	BGIOSGA010968	Os03g0283200	Down	0.0134967	Glutathione S-transferase, GST
**tPEPC-L VS WT-L**						
osa00196 Photosynthesis - antenna proteins	Os09t0346500	BGIOSGA030566	Os09g0346500	Up	0.0010183	Light-harvesting complex II chlorophyll a/b binding protein 1, Lhcb1
	Os07t0577600	BGIOSGA026003	Os07g0577600	Up	6.33E-05	Light-harvesting complex I chlorophyll a/b binding protein 2, Lhca2
osa04075 Plant hormone signal transduction/ osa04016 MAPK signaling pathway – plant/ osa04626 Plant-pathogen interaction	Os07t0129200	BGIOSGA025088	Os07g0129200	Down	0.014282	Pathogenesis-related protein, PR
	Os07t0129300	BGIOSGA025089	Os07g0129300	Down	0.0083205	Pathogenesis-related protein, PR
**tPEPC-D VS WT-D**						
osa01200 Carbon metabolism	Os07t0529000	BGIOSGA024190	Os07g0529000	Up	0.000957	Isocitrate lyase, ICL
	Os05t0186300	BGIOSGA018670	Os05g0186300	Up	0.0012799	Malic enzyme, ME
	Os03t0432100	BGIOSGA010483	Os03g0432100	Down	0.0164407	Pyruvate phosphate dikinase, PPDK
osa00195 Photosynthesis	Os12t0420400	BGIOSGA037305	Os12g0420400	Down	0.0023241	Photosystem I reaction center subunit XI, chloroplastic, Psal
osa01100 Metabolic pathways (Including osa00196 Photosynthesis - antenna proteins)	Os02t0765600	BGIOSGA005550	Os02g0765600	Up	0.044098	Alpha-amylase
	Os01t0720500	BGIOSGA004351	Os01g0720500	Down	0.0031756	Chlorophyll a-b binding protein, chloroplastic, Lhcb1
	Os01t0600900	BGIOSGA003922	Os01g0600900	Down	0.0023408	Chlorophyll a-b binding protein, chloroplastic, Lhcb1
	Os02t0194700	BGIOSGA006958	Os02g0194700	Down	0.030037	Lipoxygenase, LOX

*1: Gene ID in *EnsemblPlants*

*2: The Rice Annotation Project Database

### Gene expression analysis

**Fig. 3:**
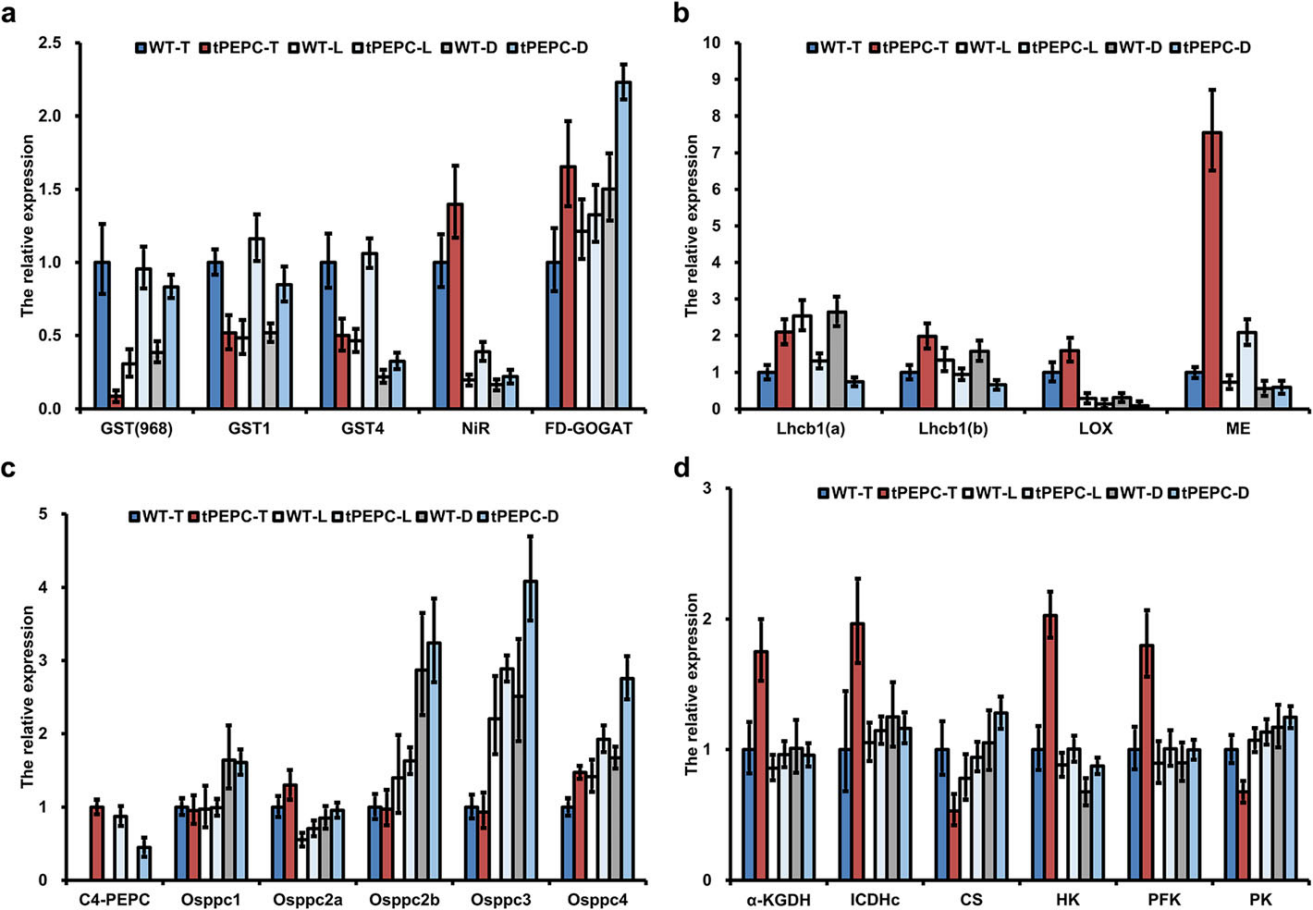
Analysis of gene expression by qRT-PCR. **a** Expression of GST, NiR and FD-GOGAT. GST (968): BGIOSGA010968. **b** Expression of Lhcb, LOX and ME. **c** Expression of *C4-PEPC* and endogenous *PEPC* genes including *Osppc1*, *Osppc2a*, *Osppc2b*, *Osppc3* and *Osppc4*. **d** Expression of genes involved in the TCA cycle (*α-KGDH*, *ICDHc* and *CS*) and glycolysis (*HK*, *PFK* and *PK*)

Proteomic analysis indicated significant differences between tPEPC and WT. To complement the changes at the transcriptional level, the gene expression of several DEPs was analyzed using quantitative real-time PCR (qRT-PCR). *GST* (968), *GST1* and *GST4* were remarkably down-regulated in tPEPC-T plants compared with WT-T, which was consistent with the regulation of expression at the protein level. However, the expressions of *GST* (968), *GST1* and *GST4* were up-regulated in tPEPC-L and tPEPC-D, which were also higher than those in WT-L and WT-D, respectively. In contrast with WT plants, the ferredoxin-nitrite reductase (*NiR*) and *FD-GOGAT* genes involved in nitrogen assimilation were up-regulated in tPEPC plants under different nitrogen source concentrations (Fig. 3a). The expression of *Lhcb1* and *Lox* genes were higher in tPEPC-T plants than in WT-T, but they were down-regulated in tPEPC-L and tPEPC-D, which were lower than that in WT-L and WT-D, respectively. The *ME* gene was up-regulated in tPEPC plants, especially in tPEPC-T (Fig. 3b). The results indicated that the expression patterns of genes involved in GSH metabolism, nitrogen assimilation and carbon metabolism in tPEPC plants were different from that in WT plants.

To investigate whether the introduction of *C*_*4*_*-PEPC* affected the expression of endogenous *PEPC* genes, we examined them using qRT-PCR. First, we analyzed the expression of *C*_*4*_*-PEPC* and found that it was gradually down-regulated in tPEPC-L and tPEPC-D. There was no obvious difference in the expression of *Osppc1* and *Osppc2a* between tPEPC and WT plants. *Osppc2b* and *Osppc3* were up-regulated under low nutrient and nitrogen deficiency, especially in tPEPC-L and tPEPC-D. *Osppc4* was up-regulated in tPEPC plants and it was significantly up-regulated in tPEPC-D (Fig. 3c). These results suggested that the expression of some endogenous *PEPC* genes indeed changed in tPEPC plants. In C_3_ plants, PEPC was mainly involved in the anaplerotic reaction of the TCA cycle, which is also in connection with glycolysis. Therefore, we analyzed the expression of rate-limiting enzyme genes in the TCA cycle and glycolysis. The expression of *α-ketoglutarate dehydrogenase* (*α-KGDH*), *isocitrate dehydrogenase* (*ICDHc*), *hexokinase* (*HK*) *and PFK* genes was higher in tPEPC-T compared with WT-T, but was no different between tPEPC-L and WT-L or tPEPC-D and WT-D. On the contrary, the expression of *CS* and *PK* genes were down-regulated in tPEPC-T compared with in WT-T (Fig. 3d). The results indicated that these TCA cycle and glycolysis genes had different expression patterns in tPEPC relative to WT under different nitrogen source concentrations.

### Enzyme activity assay

**Fig. 4:**
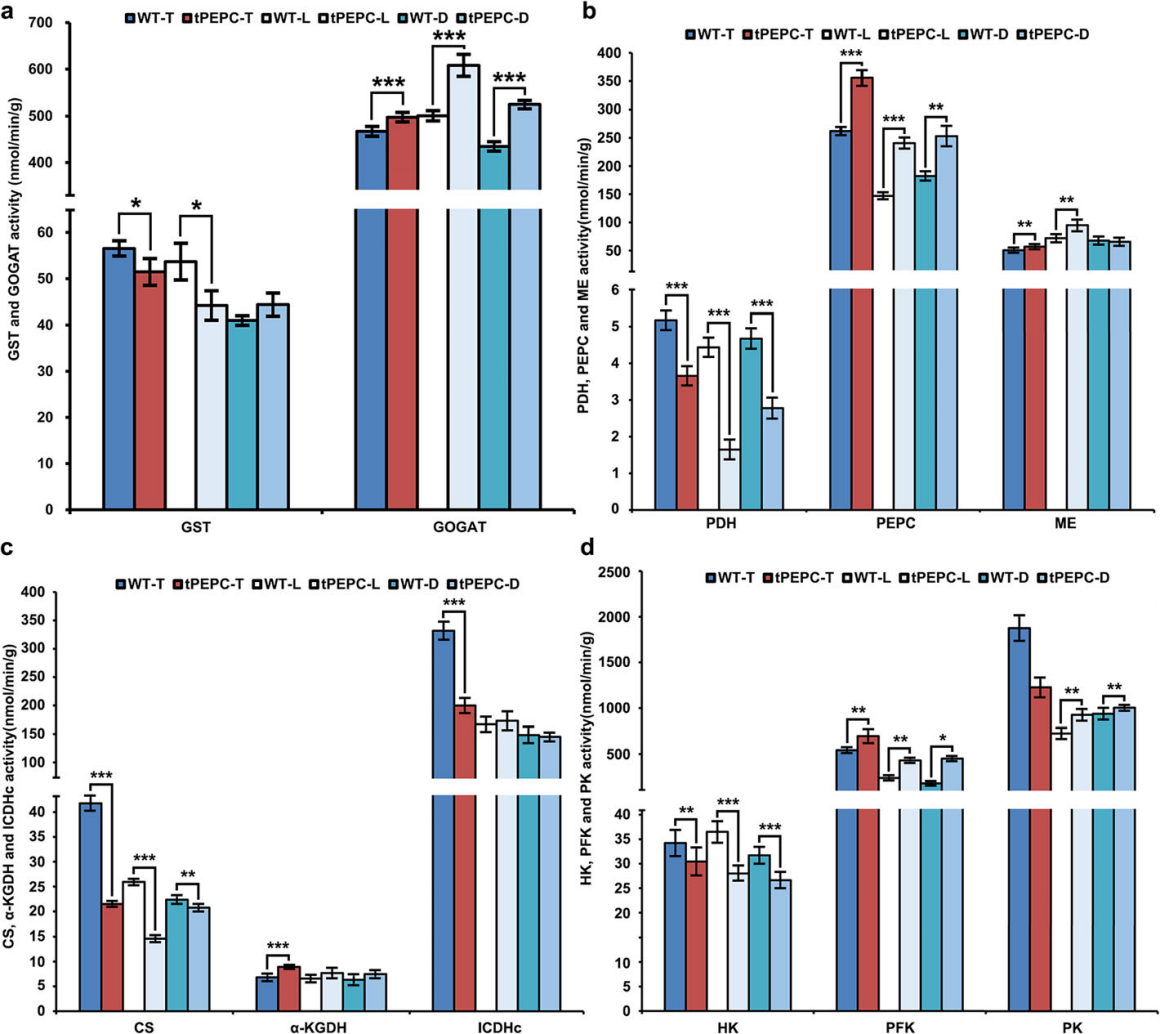
Determination of enzyme activity. **a** Enzyme activity of GST and GOGAT. **b** Enzyme activity of PDH, PEPC and ME. **c** Enzyme activity of CS, α-KGDH and ICDHc. **d** Enzyme activity of HK, PFK and PK. (*P ≤ 0.05, **P ≤ 0.01, ***P ≤ 0.001)

Given the changes in the proteome and gene expression, we conjectured a change in enzyme activity. Therefore, we measured the enzyme activity in tPEPC and WT plants. GST activity in tPEPC-T and tPEPC-L was lower than that in WT-T and WT-L, respectively. Meanwhile, GOGAT activity in tPEPC plants was higher than in WT plants under different nitrogen source concentrations (Fig. 4a). Compared with under total nutrients, PEPC activity and pyruvate dehydrogenase (PDH) activity significantly decreased under low nutrient and nitrogen deficiency in both tPEPC and WT plants. PEPC activity was higher in tPEPC plants than in WT plants, while PDH activity showed inverse activity. ME activity was higher in tPEPC-T and tPEPC-L than in WT-T and WT-L, respectively (Fig. 4b). This indicated that the activity of these enzymes correlated in response to TCA cycle changes in tPEPC plants. CS activity decreased under low nutrient and nitrogen deficiency and was significantly lower in tPEPC plants than in WT plants. The activity of α-KGDH was higher and ICDHc activity was lower in tPEPC-T than in WT-T, but there was no difference in activity between tPEPC-L and WT-L, tPEPC-D and WT-D (Fig. 4c). In addition, PFK activity was higher in tPEPC plants compared to in WT plants under different nitrogen source concentrations. On the contrary, HK was lower in tPEPC plants, especially in tPEPC-L and tPEPC-D plants. PK activity in tPEPC-T plants was lower than that in WT-T plants, whereas it was higher in tPEPC-L and tPEPC-D plants (Fig. 4d). Together, these results suggested that the activity of rate-limiting enzymes in the TCA cycle and glycolysis changed to some extent in tPEPC plants and there were significant changes in CS, PFK and PK activity.

### Analysis of organic acid content

**Fig. 5:**
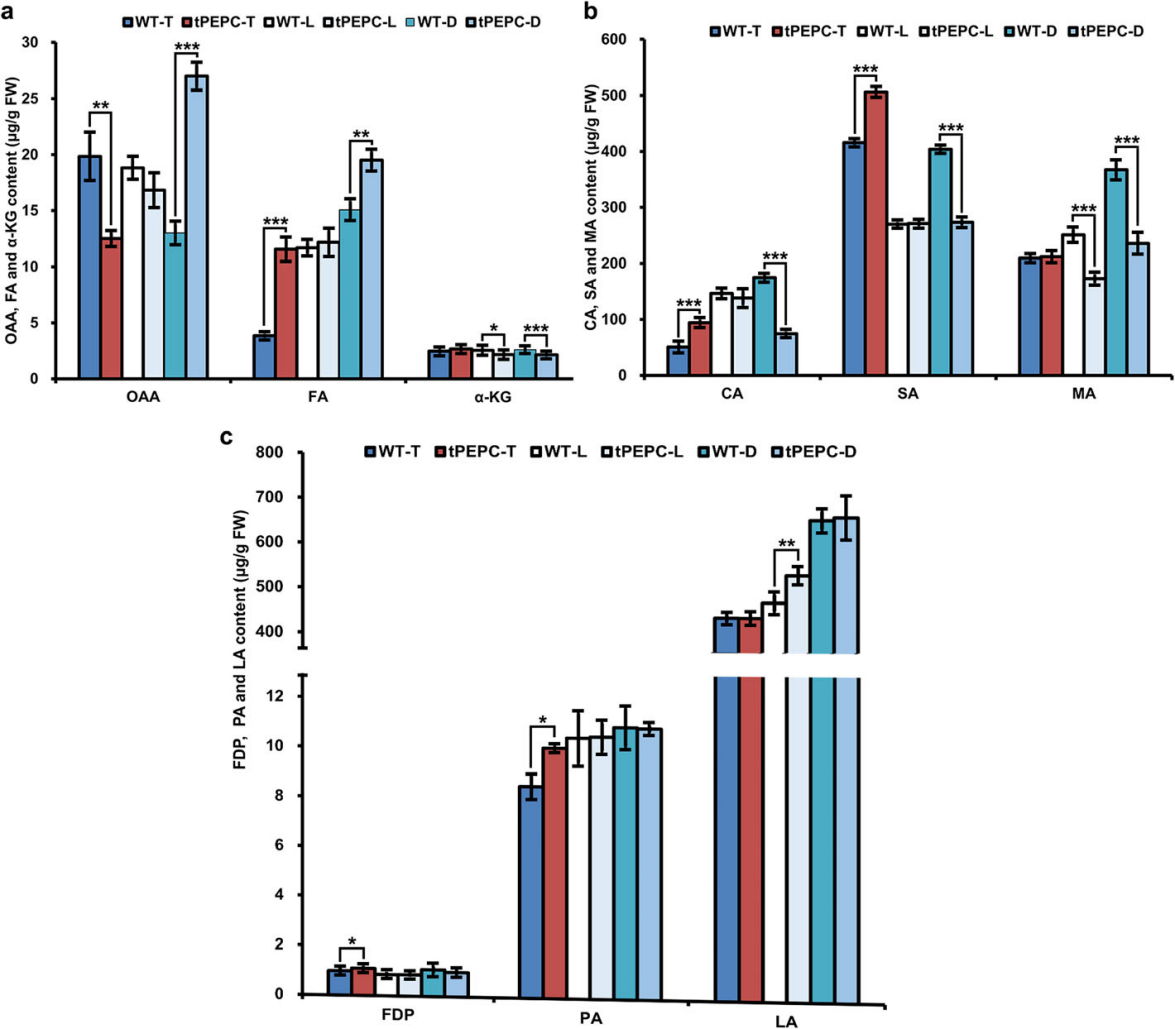
Determination of organic acid content. **a** OAA, FA and α-KG content. **b** CA, SA and MA contents. **c** FDP, PA and LA content. (*P ≤ 0.05, **P ≤ 0.01, ***P ≤ 0.001)

Subsequently, we considered whether the content of metabolic intermediate products in the TCA cycle and glycolysis changed correspondingly. We further measured organic acid content in tPEPC and WT plants. Compared with that in WT plants, OAA content was lower in tPEPC-T, while it was significantly higher in tPEPC-D. With the decrease of nitrogen concentration, the OAA contents decreased in WT plants, while they increased in tPEPC plants. The fumaric acid (FA) content in tPEPC-T and tPEPC-D was higher than that in WT-T and WT-D plants. The levels of α-ketoglutaric acid (α-KG) and malic acid (MA) were lower in tPEPC-L and tPEPC-D than in WT-L and WT-D. The citric acid (CA) and succinic acid (SA) content in tPEPC-T were significantly higher than in WT-T, but they were lower in tPEPC-D than in WT-D (Fig. 5a and b). These results indicated that the levels of these organic acids in the TCA cycle changed. Moreover, the changing trends in organic acids in tPEPC plants differed from those in WT plants under different nitrogen source concentrations. In addition, the levels of fructose-1,6 diphosphate (FDP) and pyruvic acid (PA) in tPEPC-T were higher than in WT-T, and lactic acid (LA) levels were higher in tPEPC-L plants (Fig. 5c). There were no differences between the other two comparative groups, which suggested little change in the content of these organic acids in glycolysis in tPEPC plants.

### Changes in GSH, amino acid and carbohydrate content

**Fig. 6:**
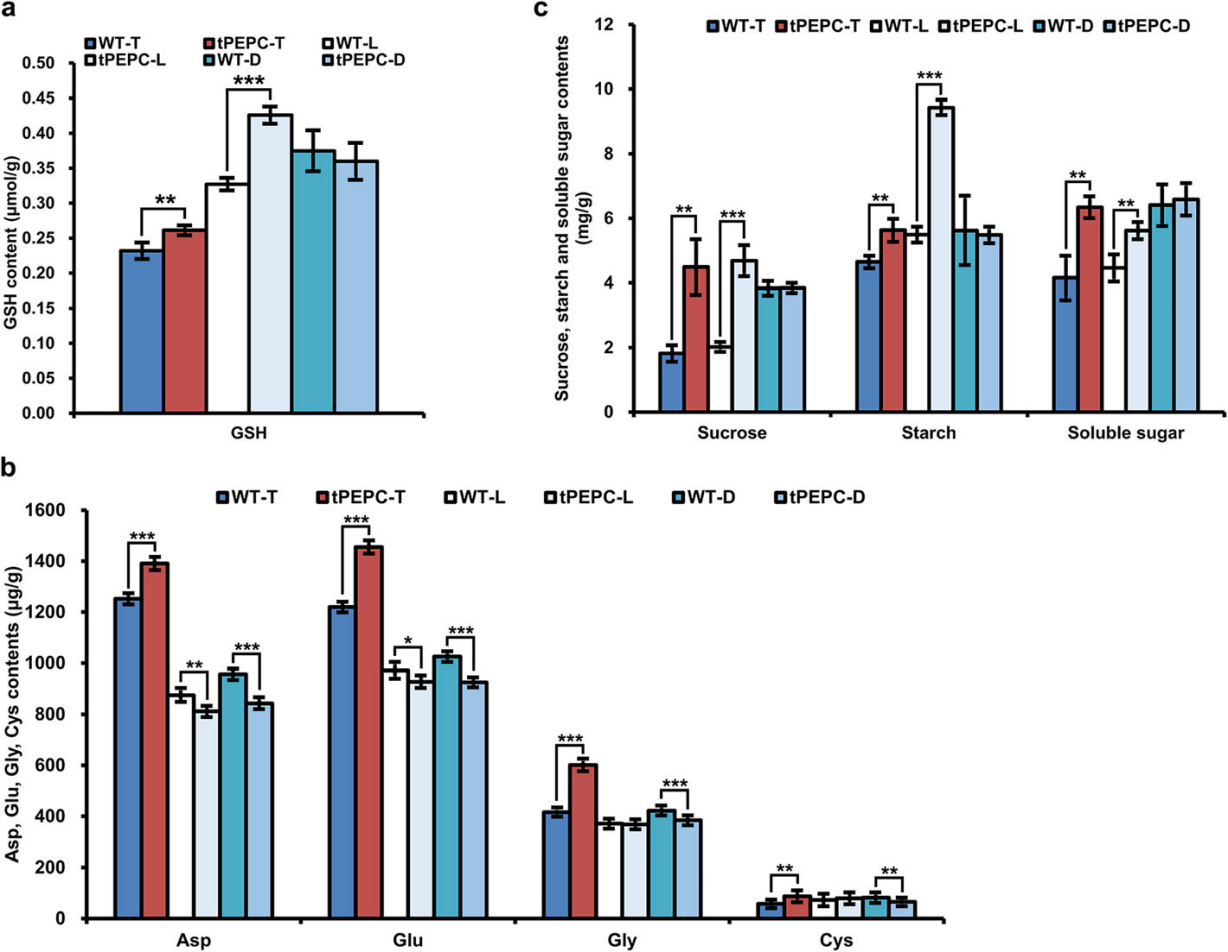
Content analysis of glutathione, amino acids and carbohydrate. **a** GSH content. **b** Asp, Glu, Gly, Cys content. **c** Sucrose, starch and soluble sugar content. (*P ≤ 0.05, **P ≤ 0.01, ***P ≤ 0.001)

In this study, GST was evidently down-regulated in tPEPC-T according to proteomic analysis. GST plays an important role in cell detoxification and catalyzes the combination of reduced GSH and toxic substances, GSH is a tripeptide synthesized from Glu, Cys and Gly. Subsequently, the content of GSH and the three amino acids were determined in tPEPC and WT plants. Compared with total nutrients, GSH content increased under low nutrient and nitrogen deficiency both in tPEPC and WT plants. Moreover, GSH levels in tPEPC-T and tPEPC-L were higher than those in WT-T and WT-L, respectively (Fig. 6a). This probably implied that GSH accumulated in tPEPC-T and tPEPC-L due to lower GST activity. Glu, Gly and Cys levels in tPEPC-T were significantly higher than in WT-T, while they were lower in tPEPC-D relative to WT-D (Fig. 6b). Additionally, OAA, an important raw material for the TCA cycle, is also the carbon skeleton of Asp and Asn. Asp levels decreased both in WT and tPEPC plants with decreasing nutrient supply. Meanwhile, Asp levels in tPEPC-T were higher than in WT-T but lower in tPEPC than in WT under low nitrogen and nitrogen deficiency (Fig. 6b). This suggested that different levels of Asp occur in WT and tPEPC plants under different nitrogen source concentrations.

As PEPC has an anaplerotic role in the TCA cycle in most non-photosynthetic tissues and C_3_ plants, it was probably the reason that the carbohydrate content changed in tPEPC plants. We determined the levels of sucrose, starch, soluble sugar in tPEPC and WT plants under different nitrogen source concentrations. The sucrose contents in tPEPC-T and tPEPC-L were about 2.5 times of that in WT-T and WT-L, respectively. Starch and soluble sugar content in tPEPC-T and tPEPC-L were significantly higher than in WT-T and WT-L. By contrast, sucrose, starch and soluble sugar content were no different between tPEPC-D and WT-D. Moreover, sucrose and soluble sugar content increased obviously in WT-D compared with WT-T, while they had a relatively higher level in tPEPC plants and there were no differences between tPEPC-D and tPEPC-T (Fig. 6c). The results indicated that these carbohydrates changed differently between tPEPC and WT with decreasing nutrient supply.

### Changes in IAA, ZT and 2iP content

**Fig. 7:**
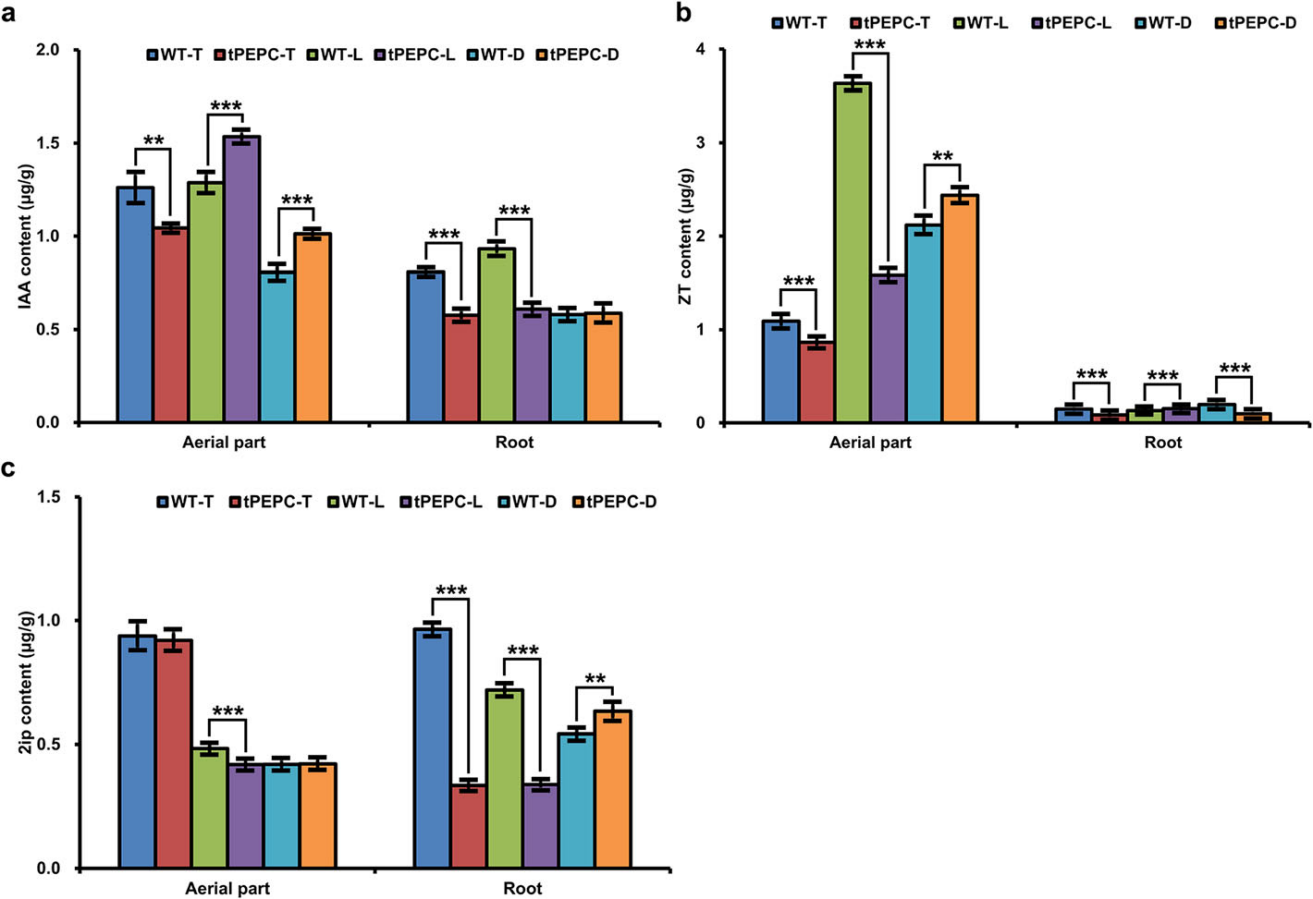
Analysis of IAA, ZT and 2iP content. **a** IAA content. **b** ZT content. **c** 2iP content. (*P ≤ 0.05, **P ≤ 0.01, ***P ≤ 0.001)

The growth and development of plants were closely related to plant endogenous hormone content. In view of the phenotypic difference between tPEPC and WT plants, we measured the levels of IAA, ZT and 2iP separately in plant aerial parts and roots. The levels of IAA were lower in both aerial parts and roots of tPEPC-T compared with WT-T. While, the levels of IAA were higher in the aerial part of tPEPC-L and tPEPC-D compared with WT-L and WT-D, respectively. The level of IAA was lower in the roots of tPEPC-L compared with WT-L, but there was no change in the IAA level in roots between tPEPC-D and WT-D (Fig. 7a). The ZT content increased in the aerial parts of tPEPC and WT under low nutrient and nitrogen deficiency compared with that under total nutrient. The levels of ZT were lower in the aerial parts of tPEPC-T and tPEPC-L compared with WT-T and WT-L, respectively, but higher in the aerial parts of tPEPC-D than in WT-D. In roots, the levels of ZT were lower in tPEPC-T and tPEPC-D than in WT-T and WT-D, and higher in tPEPC-L than in WT-L (Fig. 7b). Both in aerial parts of tPEPC and WT, the levels of 2ip under low nutrient and nitrogen deficiency were about half of those under total nutrients, and lower in the aerial parts of tPEPC-L than in WT-L. In roots, the level of 2ip in tPEPC-T was only about 35 % of that in WT-T, in tPEPC-L it was about 47 % of that in WT-L, but higher in tPEPC-D than in WT-D (Fig. 7c). It seemed that the levels of these hormones were closely related to nitrogen levels in the culture solution. It was most likely that the different hormone levels induced phenotypic differences between tPEPC and WT plants.

## Discussion

PEPC, a key enzyme located in a branch of carbohydrate metabolism in plants, replenishes PEP for the TCA cycle to support anabolism. In this study, transgenic rice expressing *C*_*4*_*-PEPC* displayed great changes in phenotype, gene expression, proteome, metabolites and hormone content.

Proteomic analysis indicated that GST significantly decreased in tPEPC-T plants. Correspondingly, GST activity was lower in tPEPC-T plants. GST plays an important role in cellular detoxification by conjugating tripeptide GSH (γ-glutamyl-cysteinyl-glycine) with toxic xenobiotic and oxidation products [16–22]. However, several studies have shown that transgenic rice expressing *C*_*4*_*-PEPC* were more tolerant to photooxidation and photoinhibition and had enhanced oxidative tolerance and drought tolerance via Ca^2+^, NO and saccharide responses [23–30]. It is most likely that other defense pathways differed from GST pathways in *C*_*4*_*-PEPC*-expressing transgenic rice, which is also probably affected by environmental conditions such as nitrogen supply. Notably, GST also participates in modulating plant growth and development [31]. GST interacting with far-red insensitive 219 was involved in the regulation of cell elongation and plant development [32]. Mutation of *OsGST4* remarkably inhibited primary root elongation, lateral root formation and shoot growth [15]. In our study, the level of GST was significantly decreased at the protein and transcriptional levels in *C*_*4*_*-PEPC* transgenic rice under normal conditions. Moreover, GSH, the substrate of GST and its components, Glu, Cys and Gly, accumulated in *C*_*4*_*-PEPC* transgenic rice, and the *C*_*4*_*-PEPC* transgenic plants had shorter roots and fewer crown roots, but plant height was taller and shoot length was longer. This suggests that the introduction of *C*_*4*_*-PEPC* mainly influenced the expression of GST in the roots and affected their growth. However, how *C*_*4*_*-PEPC* regulates the expression of GST and the mechanisms involved need further research.

PEPC catalyzes PEP, a central intermediate of glycolysis and HCO_3_^−^, to yield OAA and replenish the TCA cycle. When the amount of *ME* transcripts was raised, the activity of ME and PK increased in transgenic potato plants overexpressing *PEPC* [33]. In this study, the expression of *ME*, *Lox*, *α-KGDH*, *ICDHc*, *HK* and *PFK* genes were up-regulated in *C*_*4*_*-PEPC* transgenic rice. However, ME, α-KGDH and PFK activity were higher, but CS activity was lower in *C*_*4*_*-PEPC* transgenic rice than those in WT. These results indicated that the introduction of *C*_*4*_*-PEPC* induced changes in genes involved in metabolic reactions including the TCA cycle and glycolysis. In addition, the down-regulation of *PEPC* expression always resulted in changes of OAA, CA, MA and the other intermediates of the TCA cycle [5, 6]. Overexpression of endogenous *PEPC* in potato plants resulted in an increase in MA, 2-oxoglutarate (2-OG) and OAA [34]. There was a higher level of OAA in transgenic rice expressing maize *PEPC* [35]. In our study, *C*_*4*_*-PEPC* transgenic rice had higher levels of FA, CA, SA, FDP and PA, but had a lower level of OAA, which differed from the results of previous research. This is probably because metabolic reactions are a dynamic process, and the results revealed the difference in metabolic intermediate products between *C*_*4*_*-PEPC* transgenic rice and WT. Moreover, Li and Wang observed that the total soluble sugar in the leaves and grains of transgenic rice expressing maize *PEPC* significantly increased compared with those in WT [36]. In our study, sucrose, starch and soluble sugar were significantly higher in *C*_*4*_*-PEPC* transgenic rice than in WT. Taken together, our study revealed that *C*_*4*_*-PEPC* altered the TCA cycle and glycolysis, and then induced changes in intermediates and metabolites.

PEPC plays an important role in the carbon-nitrogen coupling metabolism. Carbon and nitrogen metabolism were redirected, and amino acid levels increased in *PEPC* transgenic plants [9, 34, 37–39]. PEPC promotes the accumulation of protein in maturing soybean, and there are higher PEPC activity in high-protein cultivar [40]. Tang et al. indicated that transgenic rice overexpressing maize *PEPC* were more tolerant to low nitrogen stress than WT [7]. However, the down-regulation of *PEPC* genes suppressed ammonium assimilation and subsequent amino acid synthesis [5, 6]. In our study, the total nitrogen content of transgenic rice was higher at different growth stages and under different nitrogen source concentrations, which reflected the effects of *C*_*4*_*-PEPC* on nitrogen metabolism. NiR, a type of oxidoreductase, mainly converts NO_2_^−^ to NH_4_^+^ and plays an important role during NO_3_^−^ conversion [41]. GOGAT, a key enzyme in the Gln synthetase/GOGAT cycle, plays a critical role in NH_4_^+^ assimilation [42]. The expression of the *NiR* and *FD-GOGAT* genes significantly increased and *GOGAT* activity was higher in *C4-PEPC* transgenic rice in this study. Moreover, the variations of gene expression, enzyme activity, organic acids and hormone content under different nitrogen source concentrations were different between transgenic rice and WT, which suggested there was a distinct reaction to nitrogen in *C4-PEPC* transgenic rice. Glu plays a critical role in amino acid metabolism and functions in a central signaling and metabolic role in the interface of carbon and nitrogen assimilation [43]. In this study, Glu content was significantly higher in *C*_*4*_*-PEPC* transgenic rice under total nutrients, which was probably beneficial for ammonium assimilation in the Gln synthetase/GOGAT cycle. In addition, Glu is one of the components for GSH, a substrate of GST. Therefore, it is most likely that the C_4_-PEPC effect on nitrogen metabolism is related to GSH metabolism, and the mechanism of its regulation is worth further study.

In addition, hormones are vital for plant growth and development. Auxin plays an important role in plant developmental processes including cell elongation and division, shoot elongation, vascular differentiation, the regulation of tropistic responses and establishment of apical dominance [44–46]. In addition, auxin is essential for plant root development including the formation, initiation and emergence of lateral and adventitious roots, as well as root elongation [47, 48]. Rice mutants of genes involved in auxin signaling pathways showed reduced crown roots or defective root development [49–51]. Another important phytohormone is cytokinin, which is a group of phytohormones involved in several developmental and growth processes such as circadian rhythms, root development, germination, shoot development, leaf senescence and so on [52–55]. Auxin and cytokinin interact in the regulation of root meristem activities [56, 57]. The WUSCHEL-related homeobox (WOX) gene was reported as an integrator of auxin and cytokinin signaling that regulates cell proliferation during crown root development [58, 59]. Auxin-induced termed crown rootless5 (CRL5) promotes crown root initiation through the repression of cytokinin signaling [60]. IAA is the most widespread auxin in plants, and ZT and 2ip are spontaneous and predominant cytokinins in higher plants. In this study, *C*_*4*_*-PEPC* transgenic rice showed lower IAA, ZT and 2ip levels in roots under total nutrients. Shorter primary roots and fewer crown roots in tPEPC-T plants may be owing to a lower level of these hormones in roots. Nutrients including nitrogen are also important factors influencing plant root architecture, so the roots of tPEPC-L did not show a significant difference though they also had relatively lower IAA levels in roots. In addition, it has been proposed that auxin may modulate GST gene expression or enzyme activity resulting in the alteration of redox potential and then alter gene expression and cellular development [61]. Thus, we speculate that there was an inevitable relation between the lower expression level or enzyme activity of GSTs and lower IAA level in *C*_*4*_*-PEPC* transgenic rice under total nutrients.

## Conclusions

PEPC is involved in plant carbohydrate metabolism and supports carbon-nitrogen interactions. In this study, transgenic rice expressing *C*_*4*_*-PEPC* exhibited characteristics that differed from WT plants, especially shorter primary root and fewer crown roots. Gene expression, enzyme activity, phytohormone content, several metabolic intermediates and metabolites related to the TCA cycle, glycolysis and nitrogen assimilation changed in transgenic rice under different concentrations of nitrogen source. It was remarkable that proteomic analysis revealed “GSH metabolism” as the most enriched pathway in *C*_*4*_*-PEPC* transgenic rice. The expression of *GSTs* significantly decreased at the protein and transcriptional levels. Concomitantly, GST enzyme activity was lower with the accumulation of its substrate GSH in *C*_*4*_*-PEPC* transgenic rice under normal conditions. Notably, the level of Glu that a component of GSH and a critical role in the GOGAT cycle of ammonium assimilation was higher in transgenic rice. Thus, it is most likely that PEPC regulates GST and then affects nitrogen metabolism, which provides new insight into the effect of PEPC on nitrogen metabolism.

## Methods

### Plant material

tPEPC and WT plants (indica cultivar Hang2) from Rice Research Institute, Fujian Academy of Agricultural Sciences, were cultured in total nutrient solution (Yoshida) containing a normal level of nitrogen (0.11875 g/L NH_4_NO_3_), low nitrogen solution containing trace nitrogen (0.0057 g/L NH_4_NO_3_) and nitrogen deficiency solution for 20 d, and used in this study.

### Protein extraction, identification and bioinformatics analysis

The protein of samples from three biological replicates was extracted using lysis buffer (10 mM/L dithiothreitol, 8 mol/L urea, 1 % Triton-100 and 1 % protease inhibitor cocktail). Then, the protein concentration was determined using a bicinchoninic acid kit following the manufacturer’s instructions. After trypsin digestion, the peptides were labeled using a TMT kit (Thermo) according to the manufacturer’s protocol, and they were fractionated using high pH reverse-phase high-performance liquid chromatography (HPLC). Then, peptides were separated and identified by liquid chromatography-tandem mass spectrometry (LC-MS/MS). The data of MS/MS result was processed by the Maxquant search engine (v.1.5.2.8), and the tandem mass spectra were searched using the UniProt database (https://www.uniprot.org/) concatenated with a reverse decoy database. The identified proteins were annotated and classified with GO annotation proteome derived from the UniProt-GOA database (http://www.ebi.ac.uk/GOA/). Protein metabolic pathways were annotated using the KEGG database. GO terms or KEGG pathways. *P*-values < 0.05 were regarded as significantly enriched, and hierarchical cluster were performed. The protein subcellular localization was predicted by WoLF PSORT subcellular localization prediction software.

### qRT-PCR

For qRT-PCR, cDNA was generated from the previously collected RNA using the RevertAid First Strand cDNA Synthesis Kit (Fermentas, LTU). The qRT-PCR was performed with the Fast Start Universal SYBR Green Master system (Roche, USA) on a 7500 real-time PCR system (Applied Biosystems). The relative quantification of gene expression was determined using actin gene expression as a reference and the relative quantitative method (ΔΔCT) was used to evaluate the quantitative variation. Primers used in qRT-PCR are listed in Supplementary Table S1.

### Enzyme activity determination

The enzyme activity of GST, GOGAT, PEPC, PDH, ME, CS, α-KGDH, ICDHc, PFK, HK and PK were determined by reagents kits (Suzhou Comin Biotechnology Co., Ltd.) according to the manufacturer’s protocol.

### Metabolite assay

Amino acid content was detected by HPLC (Agilent 1100). GSH, sucrose, starch and soluble sugar contents were determined by reagents kits (Suzhou Comin Biotechnology Co., Ltd.) following the manufacturer’s protocol.

### IAA, ZT and 2ip assay

IAA, ZT and 2ip contents were determined by HPLC (Agilent 1100).

### Statistical analyses

Statistical analyses were performed using one-way ANOVA or Student’s t tests. *P*-values < 0.05 were considered to indicate statistical significance. Statistical calculations were performed using Microsoft Excel 2019.

## Supplementary Information

**Additional file 1: Figure S1.** Determination of total nitrogen content. **a** Total nitrogen content at seeding stage. **b** Total nitrogen content at the tillering stage.

**Additional file 2: Figure S2.** Chlorophyll (Chl) and carotenoid (Car x) content, and root dehydrogenase activity in 6-day-old seedlings. **a** Phenotype of 6-day-old seedings, **b** Root dehydrogenase activity. **c** Chl and Car x content of leaf.

**Additional file 3: Figure S3.** GO classifications of DEPs in **a** tPEPC-T/WT-T, **b** tPEPC-L/WT-L and **c** tPEPC-D/WT-D.

**Additional file 4: Figure S4.** Subcellular localization map of DEPs in **a** tPEPC-T/WT-T, **b** tPEPC-L/WT-L and **c** tPEPC-D/WT-D.

**Additional file 5: Figure S5.** GO enrichment analysis of DEPs in **a** tPEPC-T/WT-T, **b** tPEPC-L/WT-L and **c** tPEPC-D/WT-D.

**Additional file 6: Figure S6.** KEGG enrichment analysis of DEPs in **a** tPEPC-T/WT-T, **b** tPEPC-L/WT-L and **c** tPEPC-D/WT-D.

**Additional file 7: Table S1.** Primers used in this study.

## Data Availability

The datasets used and analyzed during the current study are available from the corresponding author on reasonable request.

## Abbreviations

PEPC: Phosphoenolpyruvate carboxylase
WT: Wild type plants
DEPs: Differentially expressed proteins
GST: Glutathione S-transferase
TCA: Tricarboxylic acid
CS: Citrate synthase
PFK: 6-phosphofructokinase
PK: Pyruvate kinase
FD-GOGAT: Ferredoxin-dependent glutamate synthase
GSH: Glutathione
Glu: Glutamic acid
Cys: Cysteine
Gly: Glycine
IAA: Indoleacetic acid
ZT: Zeatin
2ip: Isopentenyladenine
OAA: Oxaloacetate
Asp: Aspartic acid
Asn: Asparagine
Chl a: Chlorophyll a
FC: Fold changes
GO: Gene Ontology
KEGG: Kyoto Encyclopedia of Genes and Genomes
qRT-PCR: Quantitative real-time PCR
ME: Malic enzyme
α-KGDH: α-ketoglutarate dehydrogenase
ICDHc: Isocitrate dehydrogenase
HK: Hexokinase
