# Supplementary material for: PEPC of sugarcane regulated glutathione S-transferase and altered carbon–nitrogen metabolism under different N source concentrations in Oryza sativa
Source: BMC Plant Biol. 2021 Jun 24;21:287. doi: 10.1186/s12870-021-03071-w (PMC8223297; doi:10.1186/s12870-021-03071-w)
Supplement: Supplementary file 1 — Additional file 1: Figure S1. Determination of total nitrogen content. a Total nitrogen content at seeding stage. b Total nitrogen content at the tillering stage. [file 12870_2021_3071_MOESM1_ESM.docx]

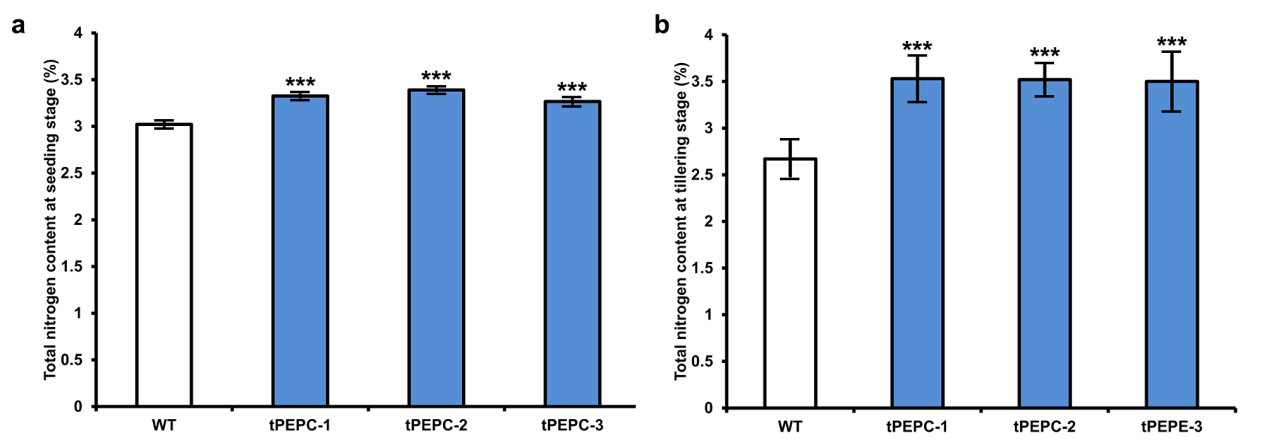


**Fig. S1.** Determination of total nitrogen content. **a** Total nitrogen content at seeding stage. **b** Total nitrogen content at the tillering stage.
