# Supplementary material for: PEPC of sugarcane regulated glutathione S-transferase and altered carbon–nitrogen metabolism under different N source concentrations in Oryza sativa
Source: BMC Plant Biol. 2021 Jun 24;21:287. doi: 10.1186/s12870-021-03071-w (PMC8223297; doi:10.1186/s12870-021-03071-w)
Supplement: Supplementary file 2 — Additional file 2: Figure S2. Chlorophyll (Chl) and carotenoid (Car x) content, and root dehydrogenase activity in 6-day-old seedlings. a Phenotype of 6-day-old seedings, b Root dehydrogenase activity. c Chl and Car x content of leaf. [file 12870_2021_3071_MOESM2_ESM.docx]

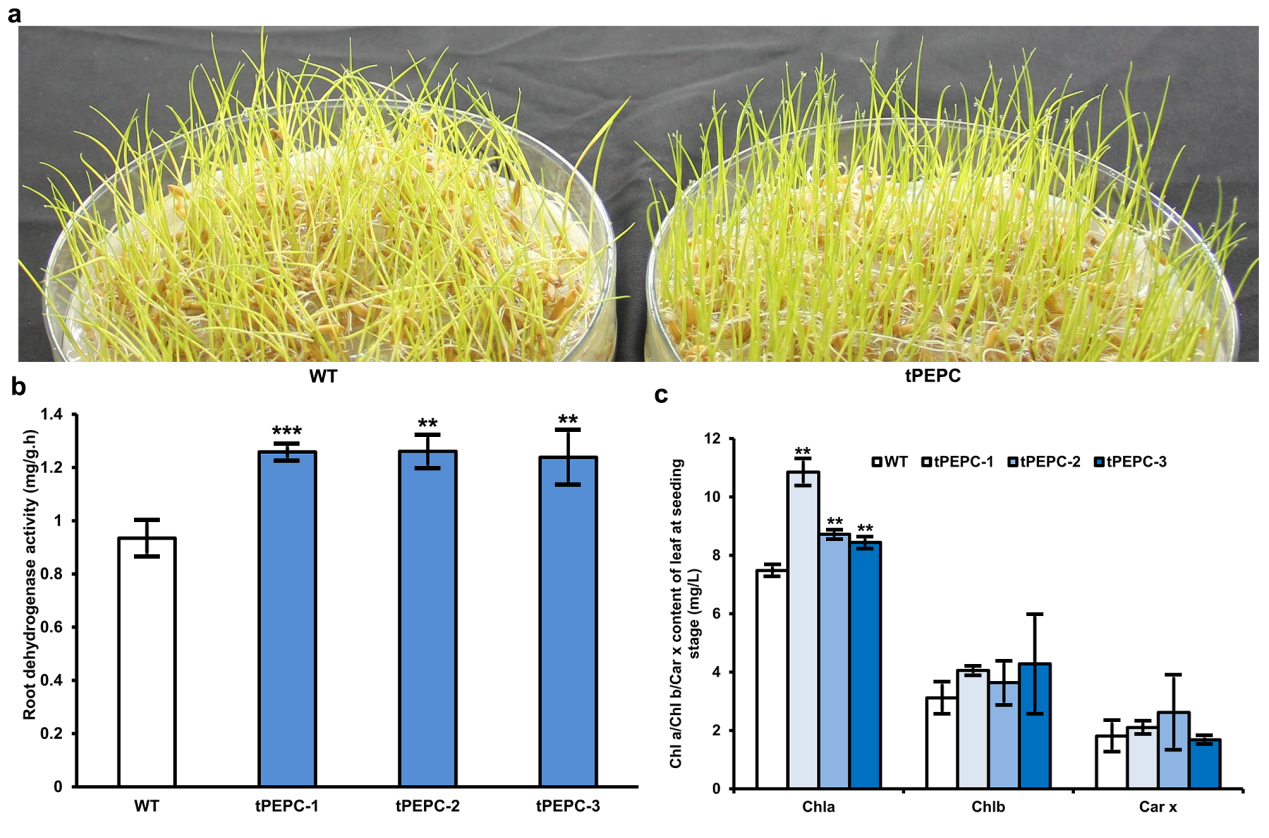


**Fig. S2.** Chlorophyll (Chl) and carotenoid (Car x) content, and root dehydrogenase activity in 6-day-old seedlings.
