# Supplementary figures and images for: PEPC of sugarcane regulated glutathione S-transferase and altered carbon–nitrogen metabolism under different N source concentrations in Oryza sativa
Source: BMC Plant Biol. 2021 Jun 24;21:287. doi: 10.1186/s12870-021-03071-w (PMC8223297; doi:10.1186/s12870-021-03071-w)

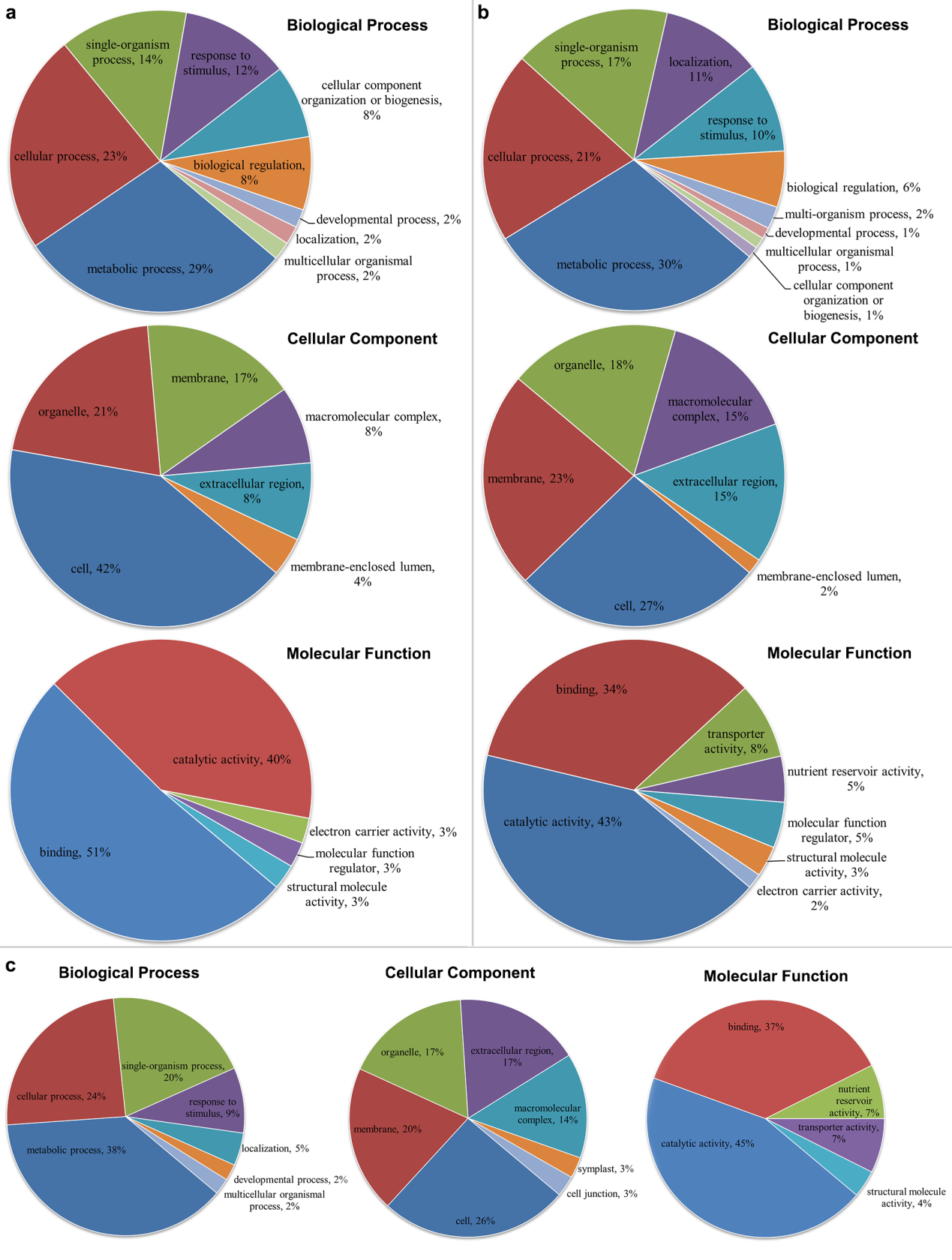


**Fig. S3.** GO classifications of DEPs in **a** tPEPC-T/WT-T, **b** tPEPC-L/WT-L and **c** tPEPC-D/WT-D.

Supplement: Supplementary file 3 — Additional file 3: Figure S3. GO classifications of DEPs in a tPEPC-T/WT-T, b tPEPC-L/WT-L and c tPEPC-D/WT-D. [file 12870_2021_3071_MOESM3_ESM.docx]

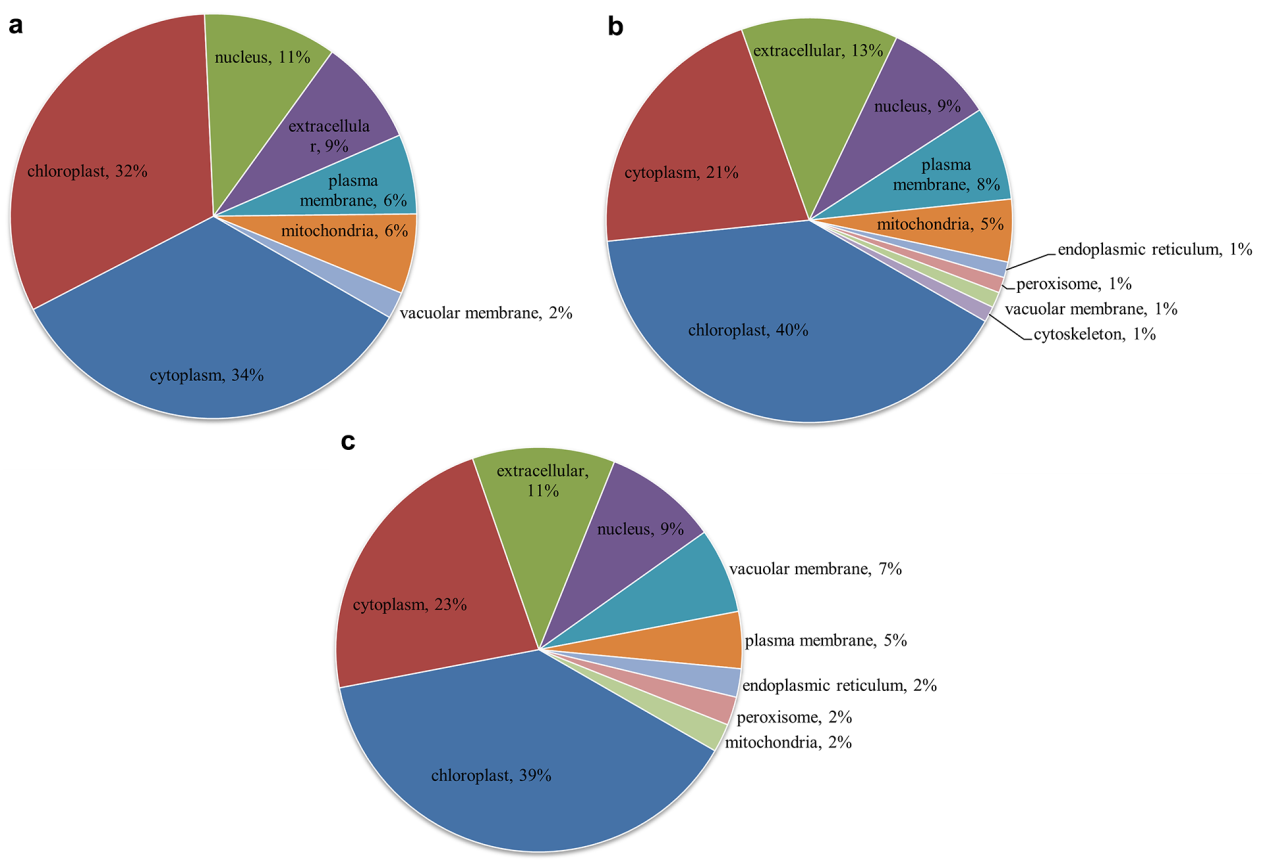


**Fig. S4.** Subcellular localization map of DEPs in **a** tPEPC-T/WT-T, **b** tPEPC-L/WT-L and **c** tPEPC-D/WT-D.

Supplement: Supplementary file 4 — Additional file 4: Figure S4. Subcellular localization map of DEPs in a tPEPC-T/WT-T, b tPEPC-L/WT-L and c tPEPC-D/WT-D. [file 12870_2021_3071_MOESM4_ESM.docx]

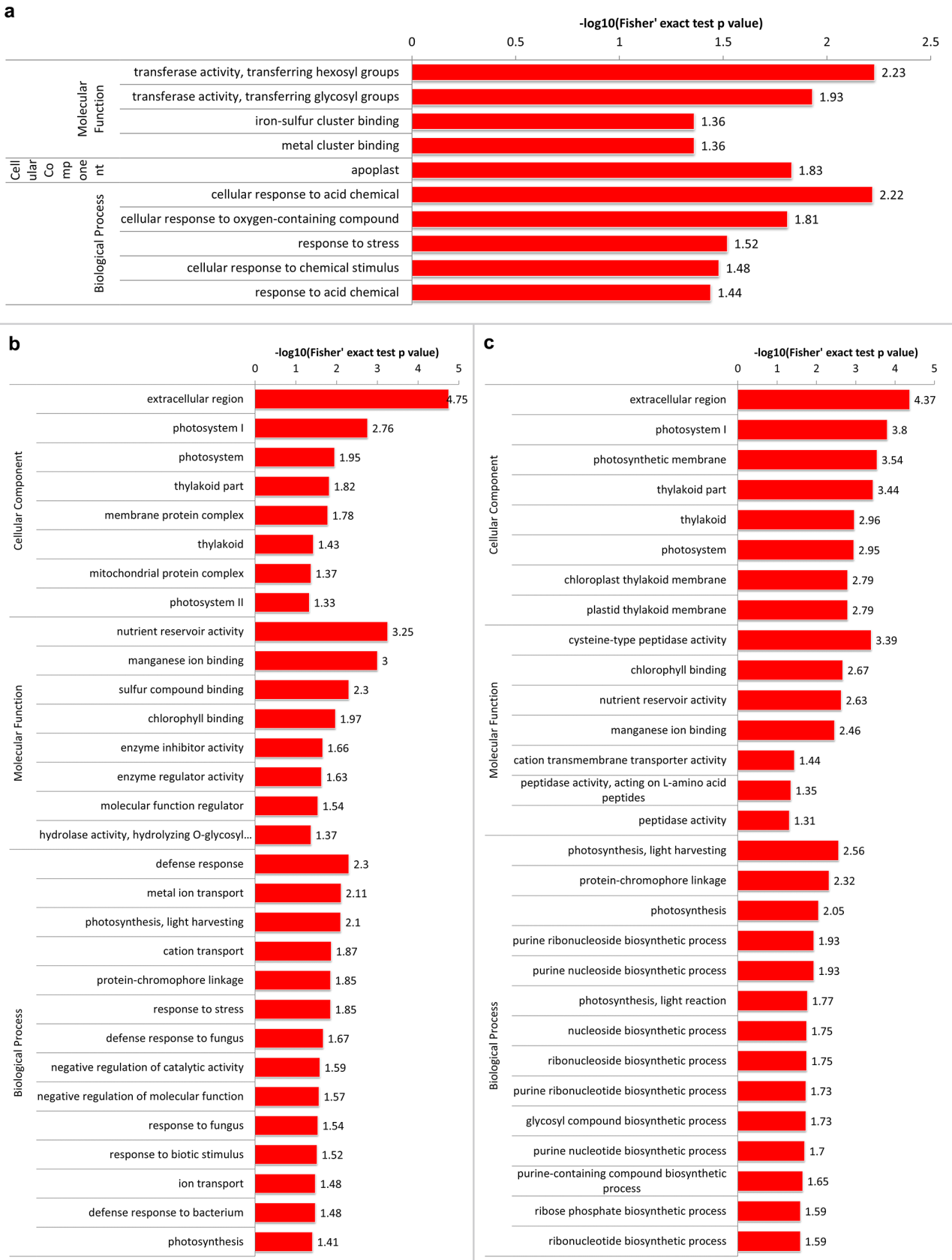


**Fig. S5.** GO enrichment analysis of DEPs in **a** tPEPC-T/WT-T, **b** tPEPC-L/WT-L and **c** tPEPC-D/WT-D.

Supplement: Supplementary file 5 — Additional file 5: Figure S5. GO enrichment analysis of DEPs in a tPEPC-T/WT-T, b tPEPC-L/WT-L and c tPEPC-D/WT-D. [file 12870_2021_3071_MOESM5_ESM.docx]

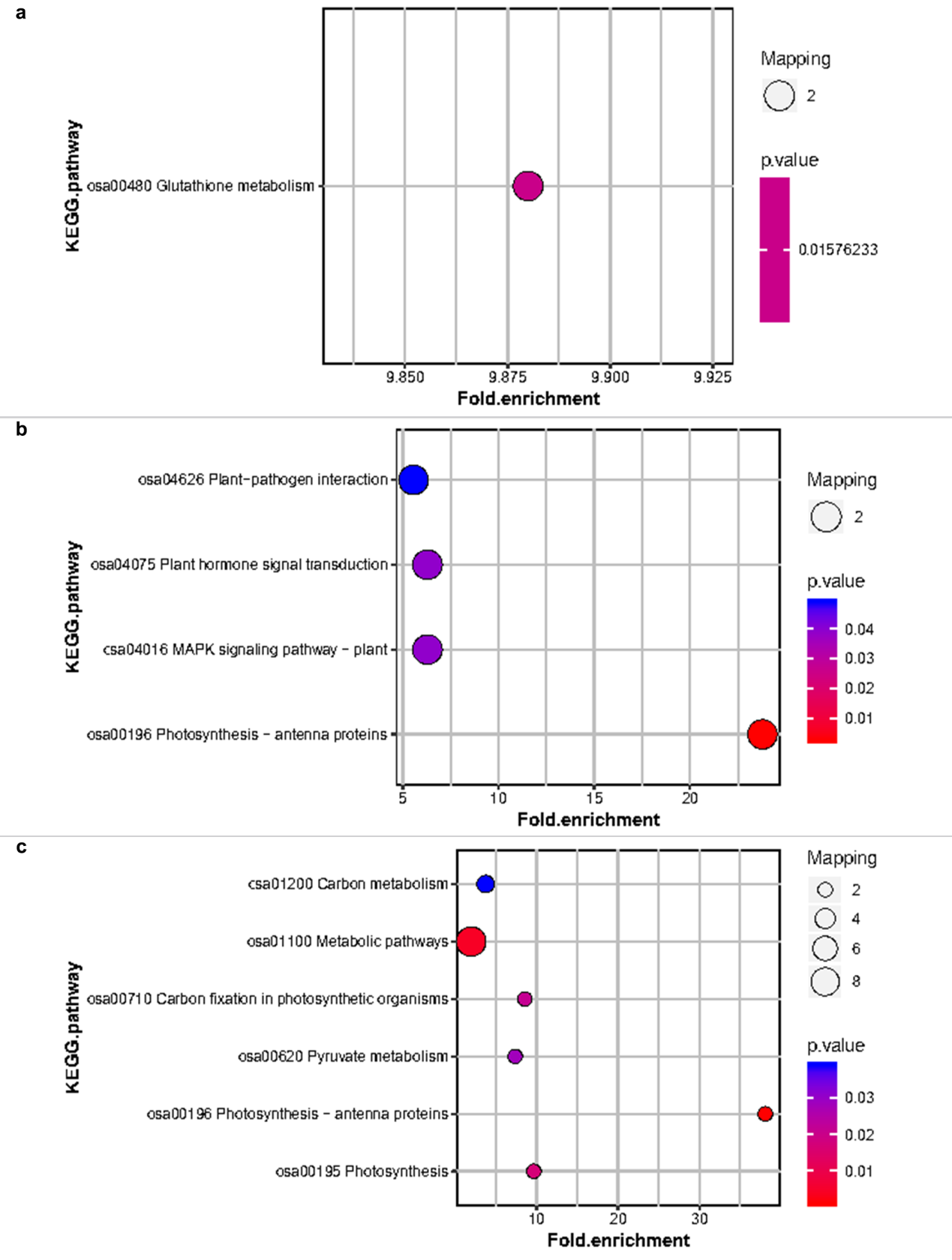


**Fig. S6.** KEGG enrichmentanalysis of DEPs in **a** tPEPC-T/WT-T, **b** tPEPC-L/WT-L and **c** tPEPC-D/WT-D.

Supplement: Supplementary file 6 — Additional file 6: Figure S6. KEGG enrichment analysis of DEPs in a tPEPC-T/WT-T, b tPEPC-L/WT-L and c tPEPC-D/WT-D. [file 12870_2021_3071_MOESM6_ESM.docx]
