# Supplementary material for: PEPC of sugarcane regulated glutathione S-transferase and altered carbon–nitrogen metabolism under different N source concentrations in Oryza sativa
Source: BMC Plant Biol. 2021 Jun 24;21:287. doi: 10.1186/s12870-021-03071-w (PMC8223297; doi:10.1186/s12870-021-03071-w)
Supplement: Supplementary file 7 — Additional file 7: Table S1. Primers used in this study. [file 12870_2021_3071_MOESM7_ESM.docx]

**Table S1.** Primers used in this study

| **Primer names** | **Sequences (5'to3')** |
| --- | --- |
| Actin150 F | TCGTCTGCGATAATGGAA |
| Actin150 R | CTCGTTGTAGAAGGTGTGATG |
| C4-PEPC F | CCATCTGCTGGCTTCTGGAGTTTC |
| C4-PEPC R | TTGTCGCCGCAGTCACACAGTG |
| Osppc1 F | CAAGAATACAGTTAAAGGGTCTGATGGG |
| Osppc1 R | GCATGATAACTGACCTTTCAATGTTTTC |
| Osppc2a F | GCGAACTATCAACTATGAAGTGCCTG |
| Osppc2a R | CAACTCCATTATTGAAAATATCTTCCGA |
| Osppc2b F | TGCCATCTTTTTTGTATCTTTCCTTTG |
| Osppc2b R | CCTTTTATTTCAGTAATAATATACATAGGCAAAA |
| Osppc3 F | ATGGTGTCATGAATTAGTGTAGTTTGC |
| Osppc3 R | GTCTTAGTTATACGTTGCTTAGCATCCC |
| Osppc4 F | TGTAGAGAAGATTGGTGGGAGTGGA |
| Osppc4 R | CACTGCTAAAAAACAGAGATAACTGAAGAA |
| GST (968) F | ACGGCACCACCAGGCTTTACA |
| GST (968) R | CGTGCCCTGTTCGTAAACCTTCT |
| GST1 F | GGCGAGGTTCTGGGCGGACT |
| GST1 R | ACGAACCCGAGCCTGCCG |
| GST4 F | CGAGGTCGTTCCGATGAGCAG |
| GST4 R | CAGGAACTCTGGTTTGTACTTGCGAA |
| NiR F | GCTGCCAAACGGTGTGACGA |
| NiR R | GCCGCTCTGGAGGCTGGTGA |
| FD-GOGAT F | ATTTAGTGATGGAAGGACAGTAGGAGC |
| FD-GOGAT R | GCCAGTTTGTAGGTCAACCGTTATC |
| Lhcb1(a) F | GTCGCCAATGCCAAGGTGTT |
| Lhcb1(a) R | GGTGAGGTAGGACGGCGGCT |
| Lhcb1(b) F | GGCGACTACGGGTGGGACA |
| Lhcb1(b) R | GACCCCGCCTTGAACCACAC |
| Lox F | TGGCGTCAACCCCATCTGC |
| Lox R | CCTCCACCGTCACCGACTCCA |
| ME F | ACTTTGCCAACCACAATGCTTTT |
| ME R | AGTTCCACCGACCACCTTGAGCG |
| CS F | GCATCTGATCTTGATCTCAAGTCCC |
| CS R | CCAGTCATCCCTCTCATCCCACC |
| ICDHc F | GCGTCGACTTCAGACTTCACGAAGGC |
| ICDHc R | GGCCATTCTCTGACTGGACTGCAATG |
| α-KGDH F | GGGCACGATAGACGACATCAAATAC |
| α-KGDH R | AAAGGTTTGATTACCCACGGCTATC |
| HK F | TCCCCATAGACAGAGTTCCAACTTTTA |
| HK R | ATTTTGTTTGTAGAGTCCATCAGGCTC |
| PFK F | CGCCAGTGTGAGAACCAACGAG |
| PFK R | ACCAGCCTTGCTATATTACACGGTAA |
| PK F | TCGGCTACTCTGGCTGGTTCA |
| PK R | GTCGCACATCTTCCGCGTGTC |
